# Beyond Traditional Prognostic Scores: Associations of Organ Dysfunction and Metastatic Malignancy with Mortality in Fournier’s Gangrene

**DOI:** 10.3390/diseases14090347

**Published:** 2026-09-18

**Authors:** Pantelie Nicolcescu, Alina Bereanu, Ionela Mihai, Manuela Arbune, Mihai Sava, Nicolae Grigore, Adrian Hasegan

**Affiliations:** 1 Faculty of Medicine and Pharmacy, Research Centre in the Field of Medical and Pharmaceutical Sciences (ReForm-UDJ), “Dunărea de Jos” University of Galați, 47 Domnească Street, 800008 Galati, Romania; pantelie.nicolcescu@ugal.ro (P.N.); manuela.arbune@ugal.ro (M.A.); 2 Faculty of Medicine, Lucian Blaga University of Sibiu, 2A Lucian Blaga Street, 550169 Sibiu, Romania; mihai.sava@ulbsibiu.ro (M.S.); adrian.hasegan@ulbsibiu.ro (A.H.); 3 Sibiu County Clinical Emergency Hospital, 2–4 Corneliu Coposu Boulevard, 550245 Sibiu, Romania; nicolaegrigore2000@yahoo.com

**Keywords:** Fournier’s gangrene, necrotizing soft tissue infection, sepsis, FGSI, SFGSI, LRINEC, metastatic malignancy, mortality prediction

## Abstract

**Background**: Fournier’s gangrene (FG) is a severe necrotizing infection requiring urgent resuscitation, empirical broad-spectrum antimicrobials, and radical debridement. We evaluated established severity scores, Sepsis-3-defined organ dysfunction, and malignancy status in a single-center cohort with surgically confirmed FG. **Methods**: This retrospective observational study analyzed 21 consecutive adult patients treated between 1 January 2019 and 31 January 2025 at a Romanian tertiary referral center. Pre-treatment baseline FGSI, SFGSI, LRINEC, NLR, SOFA, and qSOFA were calculated. All patients received intravenous antimicrobials within 90 min and underwent surgical debridement within 6 h. In-hospital mortality was assessed via ROC curves, Fisher’s exact test, Haldane–Anscombe odds ratios (ORs), and Firth penalized logistic regression. **Results**: In-hospital mortality was 19.0% (4/21). Admission Sepsis-3 sepsis (SOFA ≥ 2) was present in 7/21 patients (33.3%) and accounted for all four deaths, whereas zero deaths occurred among the 14 patients without organ dysfunction (Fisher’s exact *p* = 0.006; OR 37.33, 95% CI 1.60–866.90). All nine qSOFA-positive patients without SOFA dysfunction (42.9%) and five patients without organ dysfunction (23.8%) survived. FGSI showed the highest discrimination (AUC 0.934, 95% CI 0.76–1.00), followed by SFGSI (AUC 0.919, 95% CI 0.78–1.00) and LRINEC (AUC 0.860, 95% CI 0.68–0.99); NLR showed poor discrimination (AUC 0.574, 95% CI 0.13–1.00). Metastatic solid malignancy was present in 2/4 non-survivors versus 0/17 survivors (Fisher’s exact *p* = 0.029; OR 35.00, 95% CI 1.27–961.40). All four non-survivors met Sepsis-3 criteria (two with and two without metastases). Seven patients required ICU admission (all four non-survivors and three survivors). **Conclusions**: Admission Sepsis-3-defined organ dysfunction was strongly associated with in-hospital mortality. FGSI and SFGSI demonstrated the highest point-estimate discrimination, LRINEC also showed good discrimination, and NLR demonstrated limited standalone discriminative value. Metastatic malignancy occurred exclusively among non-survivors but overlapped with Sepsis-3-defined sepsis. These findings support the combined assessment of Sepsis-3-defined organ dysfunction and established severity scores for early risk stratification, warranting validation in larger cohorts.

## 1. Introduction

Fournier’s gangrene is a severe and rapidly progressive necrotizing fasciitis involving the perineal, perianal, and external genital regions [1,2]. The condition was originally described by Jean-Alfred Fournier in 1883 as a fulminant gangrenous process affecting otherwise healthy young men [1]. Subsequent clinical evidence demonstrated that Fournier’s gangrene is rarely idiopathic and may originate from anorectal, genitourinary, or cutaneous infections [2]. Despite advances in diagnosis and treatment, it remains a life-threatening surgical emergency associated with substantial mortality [2,3]. Population-based studies have also demonstrated considerable morbidity and healthcare resource utilization associated with the disease [4].

Early recognition and aggressive multidisciplinary treatment are essential components of contemporary management [5,6]. Urgent surgical debridement remains the cornerstone of source control [5,6]. Hemodynamic stabilization and appropriate broad-spectrum antimicrobial therapy are also fundamental components of initial treatment [5,6]. However, early diagnosis may be difficult because initial cutaneous manifestations can be subtle and may resemble non-necrotizing soft-tissue infections [7]. Diagnostic uncertainty during the early stages can therefore delay recognition of an otherwise rapidly progressive disease [7].

Systemic deterioration in severe infection may progress to acute organ dysfunction and septic shock. Large studies and meta-analytic evidence have demonstrated the prognostic relevance of SOFA- and qSOFA-based clinical criteria in patients with suspected infection [8,9]. The Sequential Organ Failure Assessment (SOFA) score was originally developed to provide an objective assessment of organ dysfunction and failure [10]. The Third International Consensus Definitions for Sepsis and Septic Shock (Sepsis-3) subsequently defined sepsis as life-threatening organ dysfunction caused by a dysregulated host response to infection [11]. Under Sepsis-3, organ dysfunction is operationalized as an acute increase in the SOFA score of two or more points [11]. This framework provides a standardized approach to identifying clinically significant organ dysfunction in patients with infection [8,9,10,11].

Several disease-specific scoring systems have been developed to facilitate risk stratification in Fournier’s gangrene. Laor and colleagues introduced the Fournier’s Gangrene Severity Index (FGSI) using physiological and laboratory variables to estimate disease severity and mortality risk [12]. Lin and colleagues subsequently developed and validated the Simplified Fournier’s Gangrene Severity Index (SFGSI) [13]. The SFGSI uses three routinely available admission variables: serum creatinine, potassium, and hematocrit [13]. Yilmazlar and colleagues proposed the Uludağ Fournier’s Gangrene Severity Index (UFGSI), which incorporates age and disease extent in addition to physiological variables [14]. These disease-specific instruments provide different approaches to early mortality risk stratification [12,13,14].

Other laboratory-based tools and inflammatory markers have also been investigated. Wong and colleagues developed the Laboratory Risk Indicator for Necrotizing Fasciitis (LRINEC) score to distinguish necrotizing fasciitis from other severe soft-tissue infections using routinely available laboratory parameters [15]. Unlike FGSI and SFGSI, LRINEC was originally developed primarily as a diagnostic rather than a mortality-prediction instrument [15]. The neutrophil-to-lymphocyte ratio (NLR) has also been investigated as an accessible marker of systemic inflammation and immune dysregulation [16]. More recently, Yönder and colleagues proposed the Fournier’s Gangrene Mortality Index (FGMI) as an additional composite model for mortality prediction [17].

Patient-related characteristics and systemic disease severity remain important determinants of outcome. A recent systematic review identified several factors associated with mortality in Fournier’s gangrene, including comorbidity burden, sepsis, anatomical disease distribution, and other clinical characteristics [18]. The same systematic review reported an overall mortality rate of approximately 20% across the included studies [18]. These findings indicate that prognosis reflects both the severity of the local necrotizing process and the patient’s systemic response to infection [18].

Early diagnosis and timely definitive treatment are particularly important because Fournier’s gangrene can progress rapidly. A systematic review by Lewis and colleagues emphasized the importance of prompt recognition, broad-spectrum antimicrobial therapy, and urgent surgical debridement in the management of Fournier’s gangrene [19]. Repeated surgical debridement may be required when residual or progressive necrotic tissue remains after the initial procedure [19].

Romanian clinical data provide additional information regarding disease severity and outcome. Puia and colleagues evaluated 40 patients with Fournier’s gangrene treated in North-Eastern Romania [20]. Non-survivors had significantly higher FGSI values than survivors [20]. The study also identified differences in clinical and laboratory characteristics between survivors and non-survivors [20]. These findings support the relevance of admission physiological and laboratory assessment for risk stratification in Romanian patients with Fournier’s gangrene [20].

Despite the availability of several prognostic instruments, comparative evidence regarding their performance within the same clinical population remains limited. FGSI, SFGSI, LRINEC, and NLR assess different aspects of physiological disturbance and systemic inflammation. Their concurrent evaluation may therefore provide complementary information for early risk stratification. Integrating these measures with Sepsis-3-defined organ dysfunction may further characterize the relationship between disease-specific severity assessment and systemic deterioration.

Therefore, this retrospective observational cohort study aimed to compare the prognostic performance of FGSI, SFGSI, LRINEC, and NLR for in-hospital mortality in patients with surgically confirmed Fournier’s gangrene treated at a Romanian tertiary referral center. Secondary objectives were to evaluate the associations of Sepsis-3-defined organ dysfunction, ICU admission, malignancy status, and hospital length of stay with clinical outcomes. By examining these parameters within the same cohort, the study sought to provide an integrated assessment of early clinical risk stratification in Fournier’s gangrene.

## 2. Materials and Methods

### 2.1. Study Design, Setting, and Chronology

We conducted a retrospective, observational cohort study at the Department of Urology, Sibiu County Clinical Emergency Hospital, Romania. The clinical patient-inclusion period was from 1 January 2019 to 31 January 2025. Ethics approval was formally granted on 24 March 2023 (approval no. 25), and retrospective medical record abstraction and data analysis were conducted under this approval during the authorized research period (3 April 2023 to 30 December 2025). These intervals correspond to distinct administrative and clinical processes and are reported consistently across the manuscript. All consecutive adult patients admitted with surgically confirmed FG during the inclusion period were evaluated. Twenty-one patients met all eligibility criteria, and none were excluded.

### 2.2. Eligibility and Case Definition

Inclusion criteria were: (1) age ≥ 18 years; (2) intraoperative confirmation of necrotizing fasciitis involving the perineal, perianal, and/or external genital regions requiring radical debridement; and (3) availability of complete admission physiological and laboratory data required to calculate all investigated prognostic scoring systems. The diagnosis was verified intraoperatively in all cases by the direct visualization of fascial necrosis, subcutaneous thrombosis, and non-viable soft tissue. The anatomical extent of necrosis was not documented in a standardized quantitative scoring format in the historical charts and was therefore not modeled as an independent prognostic variable.

### 2.3. Presentation, Emergency Management, Surgery, and ICU Care

The interval from initial symptom onset to hospital presentation was documented for all patients: 5/21 (23.8%) presented within the first 24 h, 14/21 (66.7%) presented between 24 and 48 h, and 2/21 (9.5%) presented between 48 and 72 h. No patient presented with hemodynamic instability requiring immediate vasopressor support at admission, and no patient fulfilled Sepsis-3 criteria for septic shock at presentation.

All physiological measurements and routine diagnostic blood samples were collected immediately upon emergency department admission, strictly prior to any therapeutic interventions or surgical procedures. The first dose of empirical broad-spectrum intravenous antimicrobial therapy was administered within 90 min of hospital arrival in all 21 patients (100%). Seventeen patients (81.0%) received linezolid plus meropenem, while four patients (19.0%) received linezolid plus piperacillin/tazobactam. These regimens ensured coverage against Gram-positive pathogens (including methicillin-resistant Staphylococcus aureus), Gram-negative bacilli, and anaerobes. Regimens were subsequently refined according to intraoperative microbiological cultures and antimicrobial susceptibility profiles when available.

All patients underwent initial radical surgical debridement within 6 h of hospital admission, following the initiation of empirical broad-spectrum antimicrobial therapy and aggressive hydro electrolytic resuscitation. Because every patient in the cohort achieved this standardized source-control target within 6 h, time to initial debridement showed no between-patient variance suitable for comparative mortality modeling. Staged surgical re-explorations were performed based on operative findings and local tissue evolution. Seven patients (33.3%) required admission to the intensive care unit (ICU). This group included all four non-survivors and three survivors. ICU admission is reported descriptively as an indicator of severe disease and high treatment intensity, rather than an independent prognostic factor, given that multivariable adjustment was not feasible with four mortality events (Table 1).

### 2.4. Data Extraction and Sepsis-3 Stratification

Clinical variables extracted from electronic and physical hospital charts included age, provenance (urban vs. rural), comorbidities, malignancy classification (solid vs. hematological, metastatic vs. non-metastatic), duration of hospital stay, surgical procedures, ICU admission, admission laboratory and physiological parameters, and in-hospital mortality. Sepsis status at admission was classified according to Sepsis-3 consensus definitions [11]. Patients with confirmed infection and an acute increase in the Sequential Organ Failure Assessment (SOFA) score ≥ 2 were classified as having Sepsis-3-defined sepsis. Patients presenting with a quick SOFA (qSOFA) score ≥ 2 but an admission SOFA score < 2 were classified as “qSOFA-positive without Sepsis-3-defined organ dysfunction”. Patients with SOFA < 2 and qSOFA < 2 were categorized as having infection without organ dysfunction. Septic shock was defined per Sepsis-3 as persistent hypotension requiring vasopressors to maintain a mean arterial pressure ≥ 65 mmHg with serum lactate > 2 mmol/L despite adequate fluid loading [11].

Because pre-admission baseline laboratory records were not available for the entire cohort, a baseline SOFA score of zero was assumed for acute organ dysfunction calculations. In patients with chronic kidney disease, heart failure, or chronic respiratory disease, this operational baseline represents an assumed reference that may reflect baseline organ impairment, which is explicitly noted as a study limitation.

### 2.5. Prognostic Scoring Systems

All prognostic scores were calculated using admission data prior to definitive surgical intervention. FGSI (Fournier’s Gangrene Severity Index) was calculated using nine acute physiological and laboratory variables defined by Laor et al. [12]. The Simplified Fournier’s Gangrene Severity Index (SFGSI) was calculated using admission serum creatinine, hematocrit, and potassium, per Lin et al. [13]. The Laboratory Risk Indicator for Necrotizing Fasciitis (LRINEC) was calculated using the six biochemical parameters described by Wong et al. [15]. The neutrophil-to-lymphocyte ratio (NLR) was calculated as the ratio of absolute neutrophil count to absolute lymphocyte count from the at-admission complete blood count. SOFA and qSOFA were calculated from the same initial pre-treatment parameters and were used strictly for Sepsis-3 categorization rather than included in the comparative prognostic score models.

### 2.6. Clinical Endpoints

The primary endpoint was in-hospital mortality. The secondary endpoints were overall length of hospital stay (in days) and ICU admission rates.

### 2.7. Statistical Analysis

Continuous variables are reported as mean ± standard deviation (SD)or median with interquartile range (IQR), based on distribution. Categorical variables are expressed as absolute frequencies and percentages (*n*%). Univariate comparisons for sparse categorical tables were evaluated using two-tailed Fisher’s exact tests based on the original observed counts. For the exploratory comorbidity analysis presented. The Haldane–Anscombe continuity correction (+0.5 added to each cell) was applied uniformly to all 2 × 2 tables to provide a single, prespecified method for odds-ratio (OR) and 95% confidence-interval (CI) estimation. For other 2 × 2 analyses containing zero cells, the same correction was used to obtain finite effect-size estimates.

Prognostic discrimination for in-hospital mortality was assessed by receiver operating characteristic (ROC) curve analysis and the area under the curve (AUC). Optimal cut-off values were derived internally using the Youden index (J = sensitivity + specificity − 1). Non-parametric bootstrap resampling with 5000 replicates was used to calculate 95% confidence intervals for AUCs. Exact binomial (Clopper–Pearson) 95% confidence intervals were calculated for sensitivity, specificity, positive predictive value (PPV), and negative predictive value (NPV). These cut-offs are explicitly reported as exploratory, cohort-derived thresholds.

Univariate associations with mortality were evaluated using Firth’s bias-reduced penalized logistic regression to control small-sample bias and separation. Penalized likelihood-ratio *p*-values and profile penalized-likelihood 95% confidence intervals were computed for both dichotomized thresholds and continuous score models (per 1-point increase for FGSI, SFGSI, and LRINEC; per 10-unit increase for NLR). Multivariable regression modeling was not attempted due to the limited number of mortality events (*n* = 4).

Correlations between admission severity scores and length of hospital stay were evaluated using Spearman’s rank correlation coefficient (ρ). To account for the confounding effect of early mortality on hospital stay duration, a sensitivity analysis restricted exclusively to the 17 survivors was performed. No multiplicity adjustments were applied given the hypothesis-generating nature of the comorbidity screen. Statistical significance was set at a two-tailed *p* < 0.05.

All statistical analyses were performed using R (version 4.5.2; R Foundation for Statistical Computing, Vienna, Austria), including the packages logistf (for Fifth penalized logistic regression), pROC (for ROC/AUC analysis, boostrap confidence intervals, and Youden-index cutt-offs), and binom (for Clopper-Pearson exact confidence intervals).

### 2.8. Ethics Statement

The study protocol was approved by the Ethics Committee for Scientific Research (CECS) of Lucian Blaga University of Sibiu, Faculty of Medicine (approval no. 25, issued on 24 March 2023). All procedures adhered to institutional ethical standards and the principles of the Declaration of Helsinki. The requirement for written informed consent was waived by the ethics committee due to the retrospective study design and the use of anonymized patient records. To prevent re-identification, patient identifiers, individual table linking codes, and exact multi-variable patient profiles are omitted, and data are presented exclusively in aggregate format.

## 3. Results

### 3.1. Demographic and Clinical Characteristics

All 21 patients included in the study were male. Mean age was 66.9 ± 10.9 years, and median age was 67.0 years (interquartile range [IQR], 63.0–71.0 years). Fourteen patients (66.7%) originated from urban areas and seven (33.3%) from rural areas. In-hospital mortality was 19.0% (4/21), while 17 patients (81.0%) survived to discharge. Mortality did not differ significantly between urban (2/14, 14.3%) and rural patients (2/7, 28.6%; Fisher’s exact *p* = 0.584).

Mean hospital length of stay was 17.8 ± 8.2 days and median length of stay was 16.0 days (IQR, 12.0–20.0 days). Regarding surgical management, 1 patient (4.8%) underwent a single debridement, 11 patients (52.4%) required two procedures, and 9 patients (42.9%) underwent three staged procedures. Initial orchiectomy was performed in 13 patients (61.9%) and suprapubic cystostomy in 12 patients (57.1%). Seven patients (33.3%) required ICU admission during hospitalization, including all four non-survivors and three survivors. Baseline demographic, hospitalization, and surgical characteristics are summarized in Table 2.

### 3.2. Comorbidities and Univariate Association with Mortality

The most prevalent baseline comorbidities in the overall cohort were cardiovascular diseases (10/21, 47.6%) and cachexia/malnutrition (10/21, 47.6%)**,** followed by acute kidney injury (8/21, 38.1%), diabetes mellitus (7/21, 33.3%), and chronic kidney disease (7/21, 33.3%). Any malignant disease was present in 5/21 patients (23.8%).

In exploratory univariate comparisons, none of the evaluated clinical conditions demonstrated a statistically significant association with in-hospital mortality (all two-tailed Fisher’s exact *p* > 0.05). The comorbidity analysis was fully recalculated using a uniform Haldane–Anscombe correction for ORs and 95% CIs, while Fisher’s exact *p*-values were calculated from the original observed counts. Specifically, for bronchopneumonia, the Haldane–Anscombe OR was 11.00 (95% CI, 0.98–123.98; Fisher’s exact *p* = 0.080) (Table 3).

### 3.3. Sepsis-3 Classification and In-Hospital Mortality

According to the Sepsis-3 consensus definitions applied at admission, 7/21 patients (33.3%) met the criteria for Sepsis-3-defined sepsis (SOFA ≥ 2 under the operational baseline-zero assumption). Nine patients (42.9%) presented with qSOFA ≥ 2 but had SOFA < 2 and were classified as “qSOFA-positive without Sepsis-3-defined organ dysfunction”. Five patients (23.8%) had infection without organ dysfunction (SOFA < 2 and qSOFA < 2). No patient presented with septic shock at admission.

All four in-hospital deaths occurred exclusively within the Sepsis-3-defined sepsis group (4/7, 57.1% mortality), whereas no deaths occurred among the nine qSOFA-positive patients without SOFA-defined organ dysfunction (0/9, 0%) or among the five patients with infection without organ dysfunction (0/5, 0%). The association between admission Sepsis-3 organ dysfunction and in-hospital mortality was statistically significant (57.1% vs. 0.0%; two-tailed Fisher’s exact *p* = 0.006). Using the Haldane–Anscombe correction, the estimated odds ratio was 37.33 (95% CI, 1.60–866.90). This result demonstrates a strong association between admission organ dysfunction and fatal outcome; it does not indicate an independent causal effect because organ dysfunction is intrinsic to the Sepsis-3 definition. Sepsis-3 distribution and outcome data are summarized in Table 4.

In-hospital mortality of Sepsis-3 sepsis (4/7, 57.1%)was significant compared with other categories (0/14, 0.0%). Two-tailed Fisher’s exact test: *p* = 0.006; Haldane–Anscombe OR (95% CI): 37.33 (1.60–866.90).

Among surviving patients, those classified as qSOFA-positive without SOFA-defined organ dysfunction had a significantly longer hospital stay (median 18.0 days, IQR 16.0–20.0) than survivors with infection without organ dysfunction (median 11.0 days, IQR 11.0–14.0; Mann–Whitney U = 5.5, two-tailed exact *p* = 0.029).

### 3.4. Malignancy Status and Outcome

Malignant disease was identified in 5 of 21 patients (23.8%)**.** Two patients (9.5%) had active metastatic and solid tumors (metastatic rectal adenocarcinoma and metastatic pancreatic adenocarcinoma), two patients (9.5%) had non-metastatic solid tumors (localized colon and prostate cancers), and one patient (4.8%) had a chronic hematological malignancy (granulocytic leukemia). Both patients with metastatic solid malignancy died (2/2, 100% mortality), whereas all three patients with non-metastatic or hematological malignancy survived (0/3, 0% mortality).

Metastatic malignancy was significantly associated with in-hospital mortality (100.0% in metastatic vs. 10.5% in non-metastatic/non-malignant patients; Fisher’s exact *p* = 0.029; Haldane–Anscombe OR 35.00, 95% CI 1.27–961.40). However, both patients with metastatic malignancy also met Sepsis-3 criteria for sepsis at admission; therefore, the overlap between metastatic malignancy and Sepsis-3-defined organ dysfunction, together with the limited number of deaths, precluded multivariable assessment of an independent prognostic contribution. Aggregate malignancy distribution is detailed in Table 5.

### 3.5. Prognostic Scoring Systems and ROC Analysis

Overall admission score distributions in the cohort were as follows: mean FGSI was 6.2 ± 4.1 (median 4.0, IQR 3.0–9.0); mean SFGSI was 1.3 ± 1.3 (median 1.0, IQR 0.0–2.0); mean LRINEC was 5.6 ± 3.0 (median 6.0, IQR 4.0–8.0); and mean NLR was 16.4 ± 14.4 (median 12.0, IQR 6.5–22.4, range 2.5–60.0).

In ROC curve analysis for in-hospital mortality, FGSI demonstrated the highest point-estimate discriminatory accuracy with an AUC of 0.934 (bootstrap 95% CI, 0.76–1.00), followed by SFGSI with an AUC of 0.919 (bootstrap 95% CI, 0.78–1.00). LRINEC showed good discrimination with an AUC of 0.860 (bootstrap 95% CI, 0.68–0.99), whereas NLR showed limited standalone performance with an AUC of 0.574 (bootstrap 95% CI, 0.13–1.00). Optimal cut-offs were derived internally using the Youden index from the same dataset and are explicitly presented as exploratory thresholds. At these cohort-derived cut-offs, FGSI (≥8), SFGSI (≥2), and LRINEC (≥7) all achieved 100% sensitivity and 100% NPV, with varying specificities (76.5%, 70.6%, and 58.8%, respectively). ROC performance metrics and their corresponding 95% confidence intervals are reported in Table 6.

### 3.6. Univariate Firth Penalized Logistic Regression Analysis

To address small-sample bias and complete separation arising from the small event count (*n* = 4), univariate Firth penalized logistic regression was performed for both dichotomized exploratory cut-offs and continuous scores. At the internally derived cut-offs, FGSI ≥ 8 (OR 27.00, 95% profile penalized-likelihood CI 2.22–3857.86; *p* = 0.0077), SFGSI ≥ 2 (OR 20.45, 95% CI 1.72–2895.57; *p* = 0.0149), and LRINEC ≥ 7 (OR 12.60, 95% CI 1.08–1767.67; *p* = 0.0454) demonstrated significant associations with mortality, whereas NLR ≥ 18.8 was not significant (OR 3.00, 95% CI 0.36–25.83; *p* = 0.3852). The extremely wide confidence intervals illustrate the substantial statistical uncertainty inherent to small events (Table 7).

When modeled as continuous predictors, each 1-point increase in admission FGSI was associated with an OR of 1.62 (95% profile penalized-likelihood CI 1.16–3.45; penalized LR *p* = 0.0003). Each 1-point increase in SFGSI yielded an OR of 4.01 (95% CI 1.41–35.22; *p* = 0.0021), and each 1-point increase in LRINEC yielded an OR of 1.68 (95% CI 1.02–4.37; *p* = 0.0061). For NLR, modeled per 10-unit increase, the association did not reach formal statistical significance (OR 1.45, 95% CI 0.77–2.95; penalized LR *p* = 0.0696). Continuous Firth regression models are presented in Table 8.

### 3.7. Association Between Severity Scores and Length of Hospital Stay

In the overall cohort (N = 21), no statistically significant correlations were observed between admission severity scores and total duration of hospital stay: FGSI (ρ = −0.25, *p* = 0.27), SFGSI (ρ = −0.10, *p* = 0.67), LRINEC (ρ = −0.31, *p* = 0.18), and NLR (ρ = −0.03, *p* = 0.88).

Because in-hospital death mechanically shortens the duration of hospitalization in high-risk patients, a sensitivity analysis restricted exclusively to the 17 surviving patients was conducted. In this survivor-only subgroup, the correlations remained non-significant: FGSI (ρ = −0.114, *p* = 0.664), SFGSI (ρ = 0.222, *p* = 0.391), LRINEC (ρ = −0.092, *p* = 0.726), and NLR (ρ = −0.017, *p* = 0.948). Thus, no statistically significant association between admission severity scores and hospital length of stay was observed in either the full cohort or the survivor-only analysis. Hospital length of stay correlation metrics are detailed in Table 9.

## 4. Discussion

Fournier’s gangrene remains an aggressive surgical emergency with substantial mortality despite modern critical care and broad-spectrum antimicrobial regimens [1,2,3]. Large epidemiological investigations confirm that the disease imposes a severe clinical burden and extensive resource utilization [4]. Comprehensive reviews emphasize that early multidisciplinary management, rapid hemodynamic resuscitation, and urgent source control improve survival in specialized high-volume centers [5,6]. However, initial diagnostic delays occur frequently because early cutaneous changes often mimic benign soft-tissue infections [7,19]. Contemporary Romanian cohort data and international systematic evidence further emphasize the prognostic relevance of admission clinical severity and comorbidity burden [18,20]. National database analyses also highlight marked demographic heterogeneity and sex-specific vulnerabilities across patient populations [21]. Multicenter investigations have demonstrated the prognostic utility of FGSI- and UFGSI-based severity assessment in patients with Fournier’s gangrene [22].

The modern sepsis framework systematically redefined sepsis as life-threatening organ dysfunction resulting from a dysregulated host response to infection [11]. Meta-analytic evidence has demonstrated the prognostic relevance of SOFA-based assessment for mortality prediction in patients with sepsis [8]. Multicenter studies by Raith et al. confirmed that SOFA scores reliably predict in-hospital mortality across infected intensive care populations [9]. The original SOFA score developed by Vincent and colleagues provides an objective, quantitative assessment of progressive multi-organ failure [10]. Current international guidelines emphasize immediate antimicrobial administration and structured resuscitation as essential cornerstones of sepsis management [23]. Clinical series and reviews indicate that systemic toxicity, extensive fascial necrosis, and septic shock are associated with adverse outcomes in necrotizing soft-tissue infections [24,25,26]. Prospective clinical cohorts reaffirm that early physiological derangement and delayed presentation are strongly linked to high mortality [27].

Previous tertiary-hospital experience has demonstrated the importance of clinical severity assessment and outcome evaluation in the management of Fournier’s gangrene [28]. The Fournier’s Gangrene Severity Index (FGSI), established by Laor and colleagues, showed the highest point-estimate discriminative performance for mortality in our study [12]. Lin and colleagues developed and validated the Simplified Fournier’s Gangrene Severity Index (SFGSI), which demonstrated comparable prognostic discrimination in our cohort despite relying on only three routine admission parameters [13]. Alternative composite models, such as the Uludağ FGSI developed by Yilmazlar and colleagues, integrated patient age and anatomical dissemination to enhance predictive performance [14]. Systematic reviews and clinical evidence confirm that the Laboratory Risk Indicator for Necrotizing Fasciitis (LRINEC) score functions primarily as a diagnostic screening instrument [7,15]. Accessible inflammatory markers, such as the neutrophil-to-lymphocyte ratio (NLR), reflect the balance between innate immune activation and adaptive suppression [16]. Wong and colleagues established LRINEC using routine laboratory parameters to distinguish necrotizing fasciitis from non-necrotizing soft-tissue infections [15].

Comparative analyses by Bowen and colleagues underline the clinical utility of simplified scoring tools in time-critical emergency settings [29]. The high prognostic discrimination of SFGSI observed in our cohort aligns with clinical validation studies conducted by Tenório and colleagues [30]. Bozkurt et al. evaluated FGSI, LRINEC, and NLR in Fournier’s gangrene, further supporting comparative assessment of physiological scoring systems and inflammatory markers [31]. Large cohort studies by Azmi and colleagues similarly confirmed the robust discriminative capacity of physiological indices for in-hospital mortality [32]. Multicenter investigations by Üreyen and colleagues corroborated the enduring reference role of FGSI-based scoring models [22]. Emerging composite instruments, such as the Fournier’s Gangrene Mortality Index (FGMI) proposed by Yönder et al., aim to refine outcome prediction through integrated laboratory metrics [17].

Systematic reviews by Fernando and colleagues emphasize that LRINEC was designed as a diagnostic rather than a mortality-prediction instrument [7]. Clinical evaluations by Adli and colleagues showed that while admission severity scores correlate with mortality, they do not predict the overall duration of hospital stay [33]. Extensive single-center comparisons by Arıkan and colleagues ranked LRINEC as less accurate than disease-specific physiological scores for predicting fatal outcomes [34]. Recent evaluations by Özgul et al. indicated that leukocyte-derived inflammatory ratios show variable discriminatory capacity across different clinical settings [35]. Contemporary analyses by Kranz and colleagues highlight the urgent need for standardized clinical pathways and early risk stratification in necrotizing infections [36]. Outcome analyses by Sparenborg and colleagues validated FGSI as a predictor of both mortality and length of hospital stay in a contemporary cohort, while finding that overall comorbidity burden, including malignancy, was not independently associated with mortality [37]. Recent meta-analytic data by Seretis and colleagues confirmed that while NLR reflects systemic inflammatory burden, its standalone prognostic value in small cohorts remains heterogeneous [38]. Experimental investigations by Shareef and colleagues describe the pivotal role of toll-like receptors and inflammatory cascades in sepsis-associated acute organ injury [39,40].

### 4.1. Sepsis-3 Classification, Organ Dysfunction, and Clinical Outcomes

A primary finding of this study is the strong association between admission organ dysfunction, classified according to Sepsis-3 criteria, and in-hospital mortality [11]. All four in-hospital deaths occurred exclusively among the seven patients presenting with confirmed Sepsis-3 sepsis (SOFA ≥ 2), yielding a subgroup mortality rate of 57.1% (Fisher’s exact *p* = 0.006) [10,11]. This observation aligns with large validation studies demonstrating the strong prognostic association between SOFA-defined organ dysfunction and mortality in patients with suspected infection [8,9]. Conversely, the nine patients presenting with qSOFA ≥ 2 but SOFA < 2 experienced 100% survival, consistent with the role of qSOFA as a bedside risk-identification tool rather than an autonomous diagnostic criterion for sepsis [8,11]. However, surviving patients with initial qSOFA positivity had a significantly longer hospital stay (median 18.0 vs. 11.0 days; *p* = 0.029). Given the small subgroup sizes and the absence of standardized quantitative assessment of anatomical disease extent, this finding should be interpreted cautiously.

### 4.2. Malignancy Status and Oncological Stage

Underlying malignant disease was documented in 23.8% of the cohort. Among patients with underlying malignancy, deaths occurred only in those with disseminated solid tumors. Both patients with metastatic malignancy (pancreatic and rectal adenocarcinoma) died, demonstrating a significant association with mortality on univariate analysis (*p* = 0.029). However, both metastatic patients also presented with severe Sepsis-3 organ dysfunction, precluding multivariable modeling to establish metastatic cancer as an independent prognostic factor [11]. In contrast, all three patients presenting with localized solid tumors or chronic hematological malignancy achieved full survival following prompt surgical source control and antimicrobial therapy [5,6].

### 4.3. Dissociation Between Severity Scores and Hospital Stay

Admission severity scores demonstrated no statistically significant correlation with total hospital stay in the overall cohort or in the survivor-only sensitivity analysis (*p* > 0.05) [33]. This statistical dissociation occurs because baseline scoring systems capture acute life-threatening physiological collapse rather than the complex, prolonged requirements of secondary wound debridement, negative-pressure wound therapy, and reconstructive plastic surgery [33,34].

### 4.4. Study Strengths and Limitations

Methodological strengths include intraoperative surgical confirmation in all cases, standardized emergency management (antimicrobial therapy within 90 min and debridement within 6 h), rigorous Sepsis-3 classification, and the application of Firth’s bias-reduced penalized logistic regression to manage small-sample bias. Limitations include the retrospective single-center design, the small sample size (*n* = 21), and the low number of mortality events (*n* = 4), which yielded wide confidence intervals and precluded multivariable modeling [18,32]. Furthermore, the ROC cut-offs were internally derived using the Youden index and are susceptible to statistical optimism. Standardized quantitative scoring of anatomical necrosis extent was unavailable, and baseline SOFA scores were assumed to be zero in the absence of pre-admission records [9,10]. Therefore, all prognostic findings and derived thresholds must be interpreted as exploratory and hypothesis-generating [17,29].

## 5. Conclusions

In this retrospective single-center cohort, Sepsis-3-defined organ dysfunction at admission was strongly associated with in-hospital mortality in Fournier’s gangrene. All four deaths occurred among patients meeting Sepsis-3 criteria, whereas no deaths occurred among patients without SOFA-defined organ dysfunction, including those who were qSOFA-positive.

FGSI and SFGSI demonstrated the highest point-estimate discrimination for in-hospital mortality, while LRINEC also showed good discrimination and NLR demonstrated limited standalone discriminative value. Because the optimal thresholds were derived internally from a small cohort with only four mortality events, these cohort-specific cut-offs require external validation before clinical application.

Metastatic solid malignancy occurred exclusively among non-survivors but overlapped with Sepsis-3-defined sepsis at admission, preventing assessment of its independent prognostic contribution. All patients received empirical broad-spectrum antimicrobial therapy within 90 min and underwent initial radical surgical debridement within 6 h of hospital arrival, reflecting consistent early emergency management across the cohort.

Overall, these findings support the combined assessment of Sepsis-3-defined organ dysfunction and established severity scores, particularly FGSI, SFGSI, and LRINEC, for early risk stratification in Fournier’s gangrene. Larger multicenter prospective studies with standardized assessment of disease extent and external validation of prognostic thresholds are needed to confirm these findings and refine risk-stratification strategies.

## Figures and Tables

**Table 1:** Aggregate presentation and early-management characteristics.

Characteristic	*n*/N (%)
Presentation < 24 h from symptom onset	5/21 (23.8%)
Presentation 24–48 h	14/21 (66.7%)
Presentation 48–72 h	2/21 (9.5%)
Blood sampling and baseline scores	At admission, prior to antibiotics and surgery
First antibiotic dose	Within 90 min in 21/21 (100%)
Linezolid + meropenem	17/21 (81.0%)
Linezolid + piperacillin/tazobactam	4/21 (19.0%)
Initial debridement	Within 6 h in 21/21 (100%)
Vasopressor requirement at admission	0/21 (0%)
ICU admission	7/21 (33.3%): 4 non-survivors, 3 survivors
Quantified anatomical extent of necrosis	Not available in standardized form

Note: Individual-level linkage is intentionally omitted to protect patient anonymity in this single-center cohort.

**Table 2:** Aggregate demographic, hospitalization, surgical, and outcome characteristics (N = 21).

Characteristic	Value
Age, mean ± SD, years	66.9 ± 10.9
Age, median (IQR), years	67.0 (63.0–71.0)
Sex (Male)	21/21 (100%)
Urban provenance	14/21 (66.7%)
Rural provenance	7/21 (33.3%)
Hospital length of stay, mean ± SD, days	17.8 ± 8.2
Hospital length of stay, median (IQR), days	16.0 (12.0–20.0)
1 surgical debridement	1/21 (4.8%)
2 surgical debridements	11/21 (52.4%)
3 surgical debridements	9/21 (42.9%)
Initial orchiectomy	13/21 (61.9%)
Initial suprapubic cystostomy	12/21 (57.1%)
ICU admission	7/21 (33.3%)
In-hospital mortality	4/21 (19.0%)

Note: Individual-level data linkage is omitted to protect patient anonymity in this single-center cohort.

**Table 3:** Prevalence of comorbidities and exploratory univariate associations with in-hospital mortality.

Comorbidity	Non-Survivors (*n* = 4)	Survivors (*n* = 17)	Haldane–Anscombe OR (95% CI)	Fisher’s Exact *p*
Any diabetes mellitus	1/4 (25.0%)	6/17 (35.3%)	0.76 (0.09–6.45)	1.000
Type 2 diabetes mellitus	1/4 (25.0%)	5/17 (29.4%)	0.97 (0.11–8.44)	1.000
Chronic kidney disease	2/4 (50.0%)	5/17 (29.4%)	2.27 (0.30–17.13)	0.574
Acute kidney injury	3/4 (75.0%)	5/17 (29.4%)	5.30 (0.61–45.97)	0.253
Any cardiovascular disease	2/4 (50.0%)	8/17 (47.1%)	1.12 (0.15–8.11)	1.000
Atrial fibrillation	2/4 (50.0%)	4/17 (23.5%)	3.00 (0.39–23.36)	0.544
Chronic heart failure (NYHA III)	1/4 (25.0%)	1/17 (5.9%)	4.71 (0.37–59.79)	0.352
COPD/chronic respiratory disease	1/4 (25.0%)	1/17 (5.9%)	4.71 (0.37–59.79)	0.352
Acute or chronic respiratory failure	2/4 (50.0%)	2/17 (11.8%)	6.20 (0.68–56.18)	0.148
Bronchopneumonia	2/4 (50.0%)	1/17 (5.9%)	11.00 (0.98–123.98)	0.080
Cachexia/malnutrition	2/4 (50.0%)	8/17 (47.1%)	1.12 (0.15–8.11)	1.000

Note: OR = Odds Ratio; CI = Confidence Interval. The Haldane–Anscombe continuity correction (+0.5 added to each cell) was applied uniformly to all 2 × 2 comorbidity tables for OR and 95% CI estimation. Two-tailed Fisher’s exact *p*-values were calculated from the original observed counts. No adjustments for multiplicity were applied due to the exploratory nature of the study.

**Table 4:** Sepsis-3 classification at admission and association with in-hospital mortality.

Sepsis-3 Category	Total *n* (%)	Deaths *n* (%)	Survivors *n* (%)
Sepsis-3-defined sepsis (SOFA ≥ 2)	7/21 (33.3%)	4 (57.1%)	3 (42.9%)
qSOFA-positive without SOFA-defined organ dysfunction (qSOFA ≥ 2, SOFA < 2)	9/21 (42.9%)	0 (0%)	9 (100%)
Infection without organ dysfunction (SOFA < 2, qSOFA < 2)	5/21 (23.8%)	0 (0%)	5 (100%)
Septic shock at admission	0/21 (0%)	0	0

**Table 5:** Aggregate malignancy status and association with in-hospital mortality.

Malignancy Status	Total *n* (%)	Deaths *n* (%)	Survivors *n* (%)
Metastatic solid malignancy	2/21 (9.5%)	2 (100.0%)	0 (0%)
Non-metastatic solid malignancy	2/21 (9.5%)	0 (0%)	2 (100.0%)
Hematological malignancy	1/21 (4.8%)	0 (0%)	1 (100.0%)
No malignancy	16/21 (76.2%)	2 (12.5%)	14 (87.5%)

Mortality comparison: Metastatic (2/2, 100%) vs. non-metastatic/no malignancy (2/19, 10.5%). Two-tailed Fisher’s exact test: *p* = 0.029. Haldane–Anscombe OR (95% CI): 35.00 (1.27–961.40). Note: Tumor types are reported in aggregate without individual patient identifiers to protect confidentiality.

**Table 6:** Receiver operating characteristic (ROC) analysis and diagnostic performance of admission severity scores for in-hospital mortality.

Score	AUC Bootstrap 95% CI	Exploratory Cut-Off	Sensitivity 95% CI	Specificity 95% CI	PPV 95% CI	NPV 95% CI
FGSI	0.934 (0.76–1.00)	≥8	100% (39.8–100.0%)	76.5% (50.1–93.2%)	50.0% (15.7–84.3%)	100% (75.3–100.0%)
SFGSI	0.919 (0.78–1.00)	≥2	100% (39.8–100.0%)	70.6% (44.0–89.7%)	44.4% (13.7–78.8%)	100% (73.5–100.0%)
LRINEC	0.860 (0.68–0.99)	≥7	100% (39.8–100.0%)	58.8% (32.9–81.6%)	36.4% (10.9–69.2%)	100% (69.2–100.0%)
NLR	0.574 (0.13–1.00)	≥18.8	50.0% (6.8–93.2%)	76.5% (50.1–93.2%)	33.3% (4.3–77.7%)	86.7% (59.5–98.3%)

Note: AUC = Area Under the Curve; PPV = Positive Predictive Value; NPV = Negative Predictive Value. All cut-offs were derived internally using the Youden index and represent exploratory, cohort-specific thresholds subject to optimism. Bootstrap 95% CIs (5000 replicates) were calculated for AUCs; exact binomial (Clopper–Pearson) 95% CIs were calculated for sensitivity, specificity, PPV, and NPV.

**Table 7:** Univariate Firth penalized logistic regression for in-hospital mortality using dichotomized exploratory cut-offs.

Predictor	Odds Ratio	95% Profile Penalized-Likelihood CI	Penalized LR *p*
FGSI ≥ 8	27.00	2.22–3857.86	0.0077
SFGSI ≥ 2	20.45	1.72–2895.57	0.0149
LRINEC ≥ 7	12.60	1.08–1767.67	0.0454
NLR ≥ 18.8	3.00	0.36–25.83	0.3852

**Table 8:** Univariate Firth penalized logistic regression models using continuous score variables.

Predictor	Odds Ratio	95% Profile Penalized-Likelihood CI	Penalized LR *p*
FGSI (per 1-point increase)	1.62	1.16–3.45	0.0003
SFGSI (per 1-point increase)	4.01	1.41–35.22	0.0021
LRINEC (per 1-point increase)	1.68	1.02–4.37	0.0061
NLR (per 10-unit increase)	1.45	0.77–2.95	0.0696

Note: OR = Odds Ratio; CI = Confidence Interval; LR = Likelihood Ratio. All models were estimated using Firth’s penalized maximum likelihood estimation.

**Table 9:** Spearman rank correlations between admission severity scores and length of hospital stay in the overall cohort and surviving patients.

Score	Full Cohort (N = 21) Spearman ρ (*p*)	Survivors Only (*n* = 17) Spearman ρ (*p*)
FGSI	−0.25 (*p* = 0.27)	−0.114 (*p* = 0.664)
SFGSI	−0.10 (*p* = 0.67)	0.222 (*p* = 0.391)
LRINEC	−0.31 (*p* = 0.18)	−0.092 (*p* = 0.726)
NLR	−0.03 (*p* = 0.88)	−0.017 (*p* = 0.948)

## Data Availability

The data that support the findings of this study are available from the corresponding author upon reasonable request.

## Abbreviations

The following abbreviations are used in this manuscript:

FG	Fournier’s gangrene
SOFA	Sequential Organ Failure Assessment
qSOFA	Quick Sequential Organ Failure Assessment
SIRS	Systemic inflammatory response syndrome
FGSI	Fournier’s Gangrene Severity Index
LRINEC	Laboratory Risk Indicator for Necrotizing Fasciitis
NLR	Neutrophil-to-lymphocyte ratio
SFGSI	Simplified version of the Fournier’s Gangrene Severity Index
ICU	Intensive care unit
COPD	Chronic obstructive pulmonary disease
NYHA	New York Heart Association
ROC	Receiver operating characteristic
AUC	Area under the curve
OR	Odds ratio
CI	Confidence interval
SD	Standard deviation
IQR	Interquartile range
PPV	Positive predictive value
NPV	Negative predictive value
LR	Likelihood ratio
CECS	Ethics Committee for Scientific Research
